# Genome-Wide Identification and Functional Analysis of the Norcoclaurine Synthase Gene Family in *Aristolochia contorta*

**DOI:** 10.3390/ijms26094314

**Published:** 2025-05-01

**Authors:** Yayun Xu, Sixuan Zhang, Fanqi Meng, Wenjing Liang, Yunliang Peng, Butuo Zhu, Lili Niu, Chunling Wang, Caili Li, Shanfa Lu

**Affiliations:** 1 State Key Laboratory for Quality Ensurance and Sustainable Use of Dao-Di Herbs, Institute of Medicinal Plant Development, Chinese Academy of Medical Sciences & Peking Union Medical College, Beijing 100193, China; 2 Key Lab of Chinese Medicine Resources Conservation, State Administration of Traditional Chinese Medicine of the People’s Republic of China, Institute of Medicinal Plant Development, Chinese Academy of Medical Sciences & Peking Union Medical College, Beijing 100193, China

**Keywords:** *Aristolochia contorta*, aristolochic acid, benzylisoquinoline alkaloid, chirality, norcoclaurine, norcoclaurine synthase

## Abstract

*Aristolochia contorta* Bunge has been widely used as traditional Chinese medicine materials. However, its utility faces a great challenge due to the presence of aristolochic acids (AAs), a class of benzylisoquinoline alkaloid (BIA) derivatives. The first step in BIA skeleton formation is catalysis by norcoclaurine synthase (NCS). To gain knowledge of BIA and AA biosynthesis in *A. contorta*, genome-wide characterizations of *NCS* genes were carried out. This resulted in the identification of 15 *A. contorta NCSs*, namely, *AcNCS1–AcNCS15*. The AcNCS1–AcNCS8 proteins contained one catalytic domain, whereas the AcNCS9–AcNCS15 proteins had two. Phylogenetic analysis shows that AcNCS proteins can be classified into two clades. Gene expression analysis shows that five *AcNCSs*, including *AcNCS2*, *AcNCS4*, *AcNCS5*, *AcNCS14*, and *AcNCS15*, exhibited relatively high expression in roots and flowers, where norcoclaurine accumulated. An enzyme catalytic activity assay shows that all five of the AcNCSs can catalyze norcoclaurine formation with AcNCS14 and AcNCS15, exhibiting higher catalytic efficiency. Precolumn derivatization analysis shows that the formed norcoclaurine included (*S*)- and (*R*)-norcoclaurine, with more (*S*)-configuration. The results provide useful information for further understanding BIA and AA biosynthesis in *A. contorta* and for AA elimination and bioactive compound improvement in AA-containing medicinal materials.

## 1. Introduction

*Aristolochia contorta* Bunge belongs to the *Aristolochia* genus of the plant family Aristolochiaceae Juss. in Piperales. It is distributed in China, Korea, Russia, and Japan and has been widely used as traditional Chinese medicine (TCM) materials. *A. contorta* has anti-inflammatory, antibacterial, anti-tumor, and other pharmacological activities, and it has been used to treat various diseases, such as inflammation, asthma, and bacterial infection [1,2,3]. It is rich in bioactive compounds, such as alkaloids, terpenoids, and flavonoids [4,5,6]. Among them, alkaloids, such as magnoflorine, reticuline, glaziovine, norcorydine, isocorydine, oxonuciferine, and demethylsonodione, are an important class of bioactive compounds in *A. contorta* [7,8,9,10,11,12]. These alkaloids belong to benzylisoquinoline alkaloids (BIAs) and their derivatives. *A. contorta* is also rich in aristolochic acids (AAs), a class of BIA derivatives. They were listed as category 1 carcinogens by the International Agency for Research on Cancer (IARC) in 2008, since they can cause nephrotoxicity and carcinogenicity through induction of gene mutations in animal cells [13,14,15,16,17,18]. Therefore, the use of *A. contorta* as medicinal materials is met with great challenges and officially forbidden in China.

The elimination of AAs and increase in other bioactive compounds have become a focus of research on this plant species [11,12,19]. In order to achieve this goal, the whole genome of *A. contorta* was recently sequenced [11]. The catalytic functions of two *A. contorta* O-methyltransferases, including AcOMT1 and AcOMT2, and two cytochrome P450 proteins CYPs, including AcCYP80G7 and AcCYPQ8, were elucidated. All of them are involved in BIA biosynthesis-related pathways. AcOMT1 and AcOMT2 act as norcoclaurine 6-O methyltransferase to catalyze the conversion of norcoclaurine to coclaurine [11]. AcCYP80G7 catalyzes the conversion of reticuline to hexacyclic aporphine corytuberine [12]. AcCYP80Q8 act as a glaziovine synthase (GS) to catalyze the conversion of (*S*)- and (*R*)-N-methylcoclaurine to proaporphine alkaloid (6a*S*)- and (6a*R*)-glaziovine [12]. In addition to (*S*)- and (*R*)-N-methylcoclaurine, AcCYP80Q8 also accepts (*S*)- and (*R*)-coclaurine as substrates for the production of proaporphine alkaloid (*S*)- and (*R*)-crotsparine [12].

Norcoclaurine synthase (NCS; EC 4.2.1.78), a Pictet–Spenglerase, catalyzes the condensation of dopamine- and tyrosine-derived 4-hydroxyphenylacetaldehyde (4-HPAA) to form norcoclaurine, the central precursor of BIAs [20]. The reaction generates the isoquinoline core structure and is the first common committed step in the BIA biosynthesis pathway [21]. NCS belongs to the pathogen-related 10/Bet v (PR10/Bet v) protein superfamily [22,23]. It has a catalytic function and plays a crucial role in regulating the biosynthesis of BIAs and derivatives due to its position at the entry point of the BIA pathway [22,24]. The first *NCS* gene was isolated from *Thalictrum flavum* (*TfNCS*) based on empirical amino acid sequences of peptides [22,25,26]. Sequence comparison of *TfNCS* and *Papaver somniferum* ESTs resulted in the identification of two opium poppy *NCS* isoforms, namely, *PsNCS1* and *PsNCS2* [27]. Subsequently, *CjNCS1* was identified by screening *Coptis japonica* ESTs, and *CjPR10A* was isolated based on sequence homology with *TfNCS* [28]. So far, *NCS* genes have been identified from various plant species, such as *Argemone mexicana*, *Nelumbo nucifera*, *P. bracteatum*, *Phellodendron amurense*, *Sinopodophyllum hexandrum*, and *Stephania tetrandra* [24,29,30,31,32,33]. The results suggest that *NCS* genes belong to a multigene family. For instance, there are 11 members in *C. chinensis*, five in *Aquilegia coerulea*, and three in *Macleaya cordata* [34]. They can be classified into two clades, including NCS I and NCS II [31]. Among them, members of the NCS II clade are widely distributed across plants, such as *Citrus sinensis*, *Medicago truncatula*, *Prunus persica*, and *Cucumis sativus*. The distribution of NCS I clade members is limited to plant species producing BIAs, such as *P. somniferum*, *A. mexicana*, *Corydalis saxicola*, *Eschscholzia californica*, *C. japonica*, *T. flavum*, and *S. hexandrum* [31].

Chirality is a critical factor in the pharmacological activity of drugs. Different enantiomers often exhibit distinct biological effects [35]. For example, (−)-morphine, a well-known BIA, is a potent analgesic, whereas its enantiomer, (+)-morphine, is pharmacologically inactive [36]. This enantioselectivity underscores the importance of understanding the enzymatic mechanisms that control stereochemistry. In the BIA biosynthesis pathways, two key enzymes are responsible for the differential transformation of stereochemistry. The first is NCS, which synthesizes the core structure of BIAs. The stereochemistry of its product establishes the chiral foundation for subsequent BIA compounds [22]. The second is STORR [(*S*)- to (*R*)-reticuline], also known as reticuline epimerase (REPI) [37,38,39,40,41]. It catalyzes the stereochemical inversion of (*S*)-reticuline to (*R*)-reticuline and plays a crucial role in modulating the stereochemistry in the biosynthesis of morphinan alkaloids [37,38]. STORR is a unique protein fusion that contains a cytochrome P450 domain and an aldo–keto reductase domain [37]. The cytochrome P450 domain is responsible for the oxidation of (*S*)-reticuline to 1,2-dehydroreticuline, which is subsequently reduced to (*R*)-reticuline by the aldo–keto reductase domain [37]. The interplay among these enzymes ensures the production of enantiomerically pure BIAs, which are essential for their pharmacological efficacy. The currently reported NCS enzymes are predominantly found in Ranunculales plants, where they stereoselectively generate (*S*)-norcoclaurine [24,25,27,42,43]. However, NCS enzymes from *N. nucifera* can simultaneously produce both (*S*)- and (*R*)-norcoclaurine [44]. In addition, an NCS from the gymnosperm *Gnetum montanum* generates the majority of the products as (*S*)-norcoclaurine and a minor amount as (*R*)-norcoclaurine [44].

Although *NCS* genes have been identified in various plant species, there is no information on *NCSs* from magnoliids, including the medicinal plant *A. contorta*, which produces bioactive BIAs and carcinogenic AAs. The chirality of BIAs in *A. contorta* is also unknown. With the long-term goals of elucidating the biosynthesis pathway of BIAs and their derivatives in *A. contorta*, manipulating the accumulation of BIAs, removing AAs from AA plants, and producing *A. contorta* BIAs through synthetic biology approaches, the genome-wide identification and subsequent analysis of *A. contorta NCS* genes (*AcNCSs*) was carried out. In this study, a total of 15 *AcNCS* genes were identified and characterized. Phylogenetic analysis shows that NCSs can be classified into three clades. Among them, AcNCS proteins were included in two clades. Gene expression analysis shows that *AcNCS2*, *AcNCS4*, *AcNCS5*, *AcNCS14*, and *AcNCS15* are expressed at relatively high levels in norcoclaurine-accumulated roots and flowers. Molecular cloning and functional characterization show that all five of the AcNCS proteins analyzed can catalyze the condensation of dopamine and 4-HPAA to form (*S*)- and (*R*)-norcoclaurine. Among them, AcNCS14 and AcNCS15 exhibit high activity toward dopamine and 4-HPAA.

## 2. Results

### 2.1. Genome-Wide Identification of 15 AcNCS Genes in A. contorta

**Figure 1:**
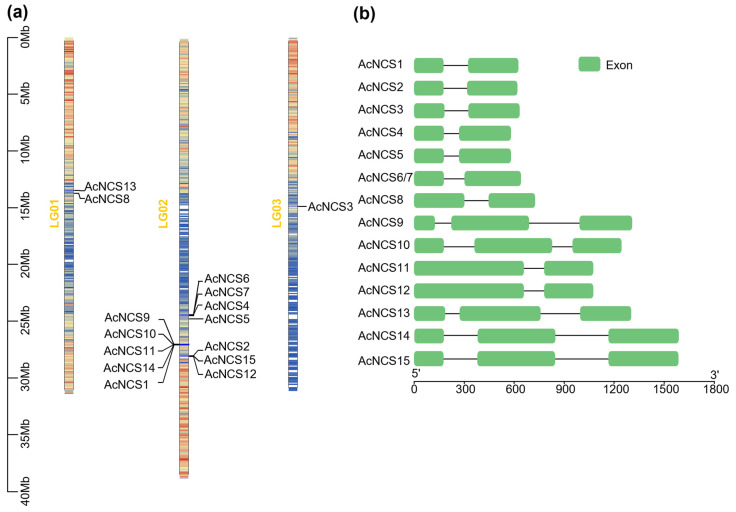
Chromosome localization and gene structure of AcNCSs: (**a**) chromosome localization of AcNCSs; (**b**) intron–exon structures of AcNCSs. Green boxes represent exons, and black lines represent introns.

In order to identify *AcNCS* genes in *A. contorta*, a tBLASTp analysis of TfNCS, PsNCS1, PsNCS2, and CjNCS2/CjPR10A proteins against the whole genome assembly of *A. contorta* was carried out [11,25,26,27,28]. Subsequent gene prediction of the retrieved genomic DNA sequences identified a total of 15 full-length members of the *AcNCS* gene family. They were designated as *AcNCS1–AcNCS15*, respectively (Table 1 and Table S1). Among them, *AcNCS6* and *AcNCS7* share 100% identity at the nucleotide and amino acid levels. Chromosome localization analysis shows that 15 *AcNCS* genes are distributed on three of the seven *A. contorta* chromosomes, including LG01, LG02, and LG03 (Figure 1a and Table 1) [11]. Among them, LG01 contains two *AcNCSs*, including *AcNCS8* and *AcNCS13*, which are clustered together. LG03 contains one *AcNCS*, namely, *AcNCS3*. LG02 contains the other twelve *AcNCSs* that are clustered at three sites, with *AcNCS4–AcNCS7* at one site; *AcNCS1*, *AcNCS9–AcNCS11*, and *AcNCS14* at the second site; and *AcNCS2*, *AcNCS12*, and *AcNCS15* at the third site (Figure 1a). Gene structure analysis shows that 10 of the 15 *AcNCSs*, including *AcNCS1–AcNCS8*, *AcNCS11*, and *AcNCS12* have an intron, whereas the other 5 have two introns (Figure 1b and Table 1). The deduced AcNCS proteins range from 160 to 355 amino acids in length (Table 1). Their molecular weights range from 17.6 to 39.0 kDa, and the isoelectric points range from 4.63 to 6.15 (Table 1).

### 2.2. Phylogenetic Relationships of AcNCS Proteins

**Figure 2:**
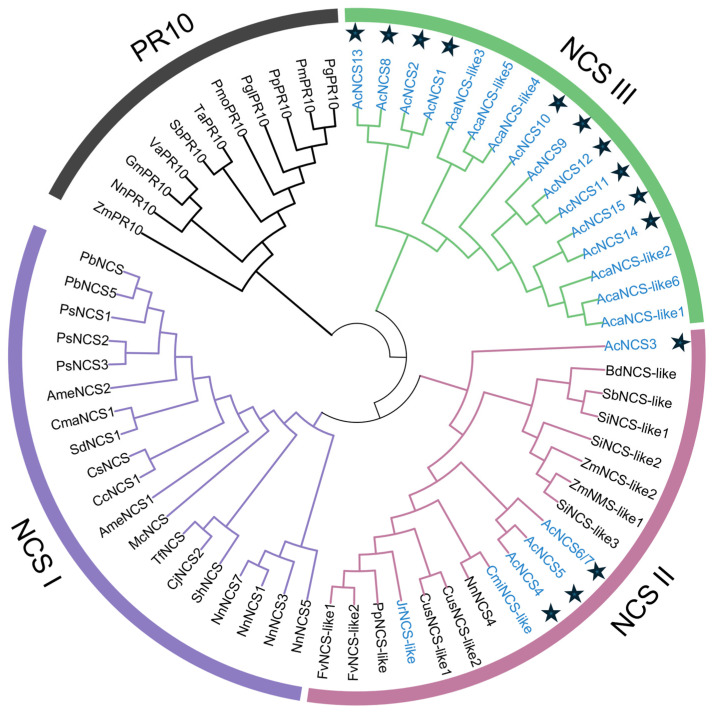
Phylogenetic relationships among NCS proteins. The neighbor-joining tree was constructed using MEGA 11 [45]. The tree consists of 55 NCSs from 22 species (Tables S2 and S3). The NCSs from Magnoliales are shown in blue. AcNCSs are marked with a five-pointed star.

In order to analyze the relationship between AcNCSs and NCSs from other plant species, as well as the classification of plant NCSs, a phylogenetic tree was constructed for 15 AcNCSs, 40 NCSs from other plants, and 11 PR10 proteins (Figure 2). The results show that these proteins can be divided into four clades, including three NCS clades and a PR10 clade. Clades NCS I and NCS II have previously been reported [31], whereas NCS III is a novel clade. AcNCSs were included in two of the three clades. Among them, five AcNCSs, including AcNCS3–AcNCS7, were included in clade NCS II, which has members that are widely distributed across plants. The other ten AcNCSs were included in the novel NCS III clade. No AcNCSs were found in clade NCS I, which has members from Ranunculales [31]. This indicates an evolutionary divergence between AcNCSs and Ranunculales NCSs.

### 2.3. Conserved Domain and Motifs of AcNCS Proteins

**Figure 3:**
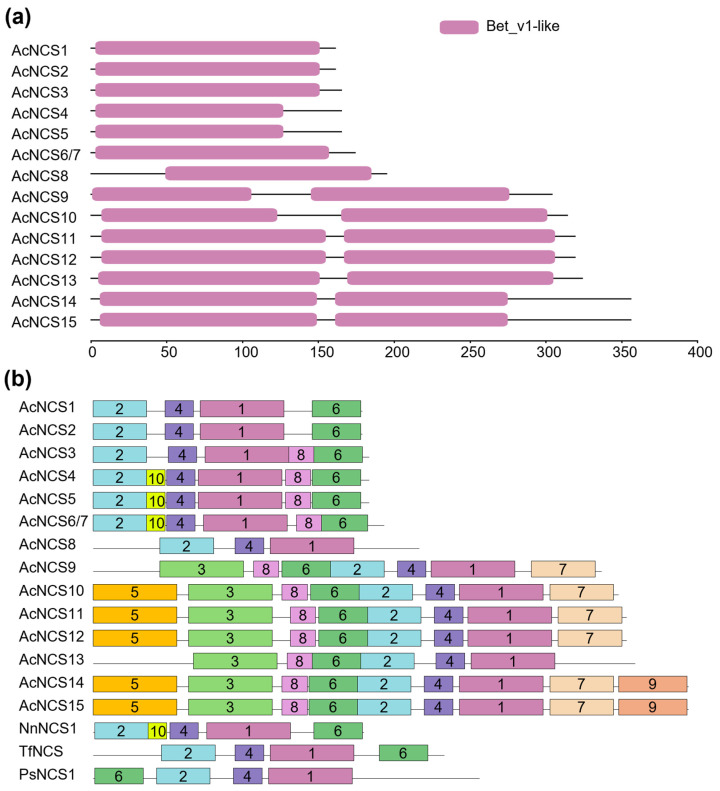
Conserved domains and motifs of AcNCS proteins: (**a**) conserved domains of AcNCS proteins; (**b**) distributions of conserved motifs of AcNCSs. Motifs predicted with MEME (https://meme-suite.org/meme/ accessed on 25 October 2024) are represented by boxes. The numbers in the boxes (1–10) represent motif 1–motif 10, respectively. The box size indicates the length of the motif. The motif consensus is shown in Table S4.

**Figure 4:**
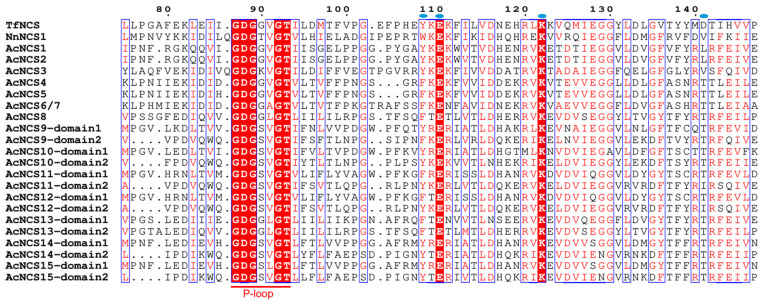
Multiple sequence alignments of AcNCSs, TfNCS, and NnNCS1. The P-loop conserved in PR10/Bet v 1 family proteins is shown [22]. The blue dots represent the four active sites (Tyr, Glu, Lys, and Asp) identified in TfNCS [46].

NCSs belong to the Bet v 1 protein family. The catalytic domain of NCS proteins, known as the Bet v 1 domain, is highly conserved in plants. In order to determine whether the identified AcNCSs contain the Bet v 1 domain, a conserved domain analysis was performed. The results show that all 15 AcNCS proteins contain the Bet v l domain (Figure 3a). Among them, AcNCS1–AcNCS8 have one Bet v 1 domain, whereas AcNCS9–AcNCS15 contain two. In addition to the conserved domains, conserved motifs could be important for the function of NCSs. To gain preliminary knowledge of the identified AcNCSs, conserved motifs were analyzed using MEME (https://meme-suite.org/meme/ accessed on 25 October 2024), with an *e*-value cutoff of 1 × 10^−10^ applied for recognition. This allowed us to identify a total of 10 conserved motifs (motifs 1–10) (Figure 3b and Table S4). The number of motifs in each NCS varied from four to nine. Among the 10 motifs, motifs 1, 2, 4, and 6, which are located in the Bet v 1 domain, were conserved in all of the NCSs analyzed. Motif 9 was specific to AcNCS14 and AcNCS15. Motif 10 was found in AcNCS4, AcNCS5, AcNCS6, AcNCS7, and NnACS1. Multiple amino acid sequence alignments of AcNCSs, TfNCS, and NnNCS1 show that all contain a P-loop conserved in PR10/Bet v 1 family proteins [22]. In addition, previous studies identified four highly conserved residues, including Tyr, Glu, Lys, and Asp, in TfNCS [46]. A sequence comparison of AcNCSs, TfNCS, and NnNCS1 shows that the Glu and Lys residues were more conserved, whereas Tyr and Asp were less conserved (Figure 4). These results indicate the conservation and diversity of AcNCSs.

### 2.4. Differential Expression of AcNCS Genes in A. contorta

**Figure 5:**
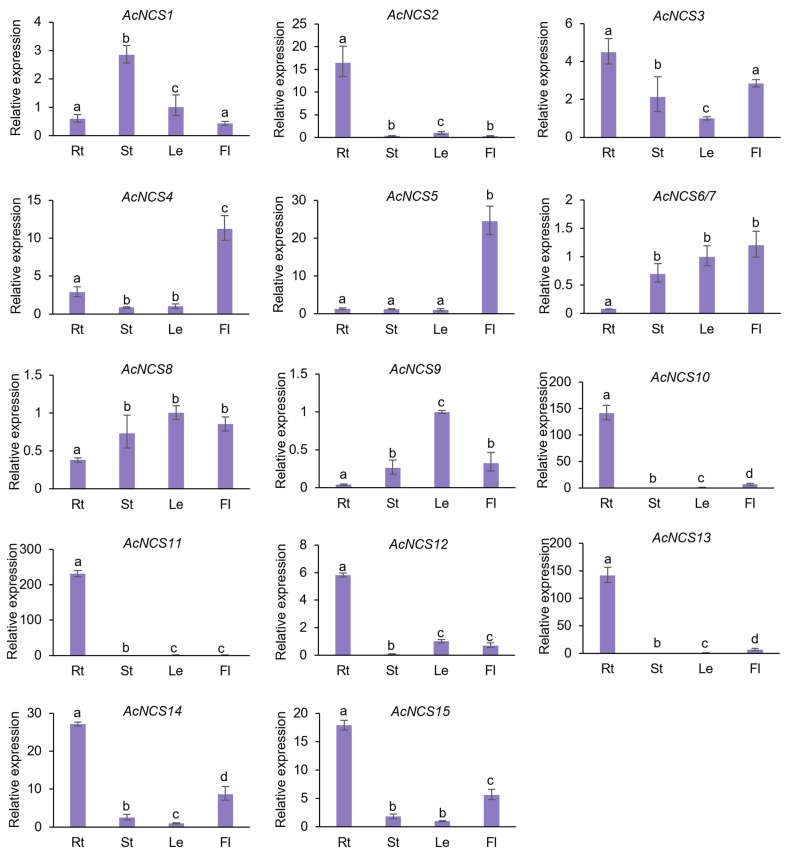
Analysis of AcNCS gene expression in roots, stems, leaves, and flowers using the qRT-PCR method. The expression level in leaves was arbitrarily set to 1. The levels in other tissues were given relative to this. Error bars represent the standard deviations of the mean value from three biological replicates. ANOVA (analysis of variance) was calculated using Graph Prism 9.0. *p* < 0.05 was considered statistically significant, which is represented by different letters above the bars. *p* ≥ 0.05 was considered statistically non-significant, which is represented by identical letters above the bars.

Gene expression patterns are usually associated with gene functions. In order to preliminarily analyze the in vivo roles of *AcNCS* genes, their expression patterns in roots, stems, leaves, and flowers of *A. contorta* were detected using quantitative real-time PCR (qRT-PCR). The results show that *AcNCS* genes were differentially expressed (Figure 5). For instance, *AcNCS1* was mainly expressed in stems, followed by leaves. *AcNCS2* and *AcNCS10–AcNCS13* were predominantly expressed in roots. *AcNCS3* was predominantly expressed in roots and flowers. *AcNCS4* exhibited relatively high expression in flowers, followed by roots. *AcNCS5* was predominantly expressed in flowers. *AcNCS6* and *AcNCS7* were mainly expressed in stems, leaves, and flowers. *AcNCS9* was mainly expressed in leaves, followed by stems and flowers. *AcNCS14* and *AcNCS15* were mainly expressed in roots, followed by flowers (Figure 5).

**Figure 6:**
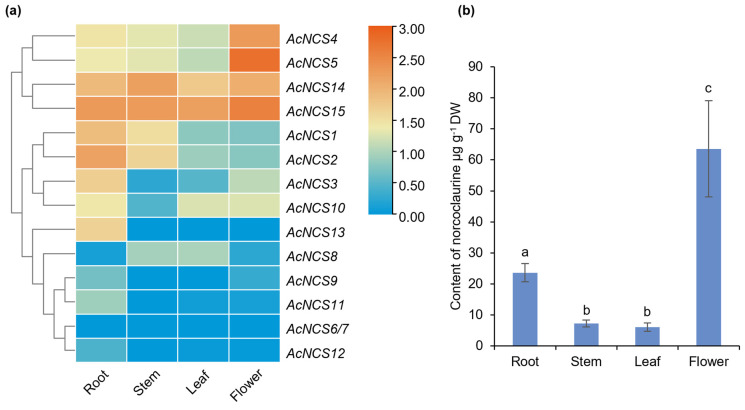
Analyses of AcNCS expression patterns and norcoclaurine contents in roots, stems, leaves, and flowers. (**a**) Expression patterns of AcNCSs in roots, stems, leaves, and flowers. The hierarchical clustering is based on the expression of AcNCSs as determined by RNA-seq [11]. (**b**) Analysis of the norcoclaurine contents in four tissues, including roots, stems, leaves, and flowers of *A. contorta*. Error bars represent the standard deviations of the mean value from three biological replicates. ANOVA (analysis of variance) was calculated using Graph Prism 9.0. *p* < 0.05 was considered statistically significant, which is represented by different letters above the bars. *p* ≥ 0.05 was considered statistically non-significant, which is represented by identical letters above the bars.

Further analysis of the gene expression using the RNA-seq data that we obtained previously shows that the *AcNCSs* expressed in roots mainly included *AcNCS1*–*AcNCS5*, *AcNCS10*, *AcNCS13*, *AcNCS14*, and *AcNCS15*. Those expressed in flowers mainly included *AcNCS4*, *AcNCS5*, *AcNCS14*, and *AcNCS15* (Figure 6a) [11]. The patterns from the RNA-seq analysis were similar to those obtained from qRT-PCR, although slight discrepancies were observed (Figure 6a). This suggests consistency between the qRT-PCR detection and RNA-seq data analysis.

### 2.5. Accumulation of Norcoclaurine in Roots and Flowers of A. contorta

NCSs catalyze the formation of norcoclaurine, the central precursor of BIAs [20]. In order to investigate the relationship between the expression of *AcNCSs* and the accumulation of norcoclaurine, we determined the contents of norcoclaurine in roots, stems, leaves, and flowers using LC-MS/MS. The results show that norcoclaurine mainly accumulates in the flowers and roots of *A. contorta* (Figure 6b). Its contents in roots, stems, leaves, and flowers were 24.61, 7.20, 6.03, and 62.56 μg g^−1^ DW, respectively. Flowers were the most abundant in norcoclaurine, followed by roots. The contents of norcoclaurine in stems and leaves were relatively low (Figure 6b). Based on the expression of *AcNCSs* and the accumulation of norcoclaurine, we speculate that AcNCS2, AcNCS4, AcNCS5, AcNCS14, and AcNCS15 could be the main AcNCS proteins responsible for norcoclaurine production in *A. contorta.*

### 2.6. AcNCS Proteins Catalyze the Formation of Norcoclaurine

**Figure 7:**
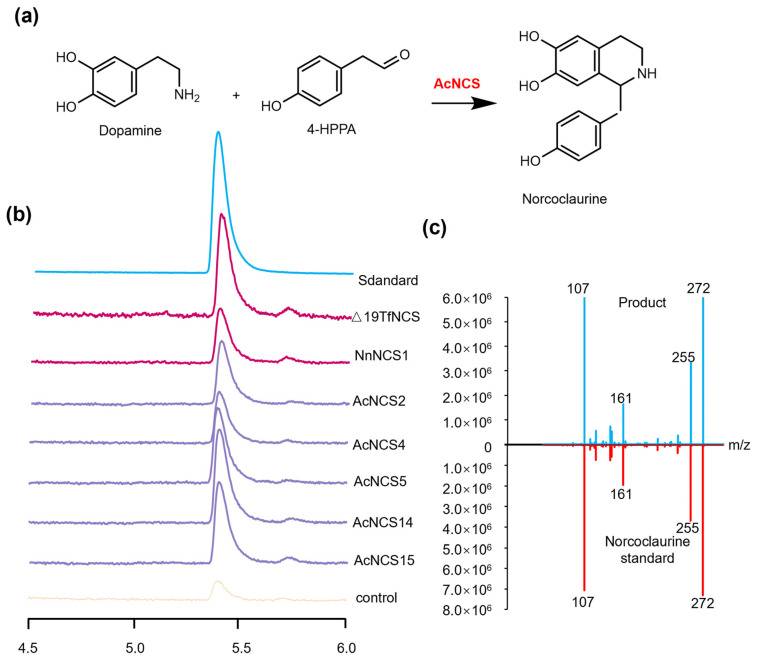
Enzymatic reaction of the AcNCSs. (**a**) Chemical reaction catalyzed by the AcNCSs. (**b**) UPLC analysis of the products from dopamine and 4-HPPA under the catalysis of AcNCSs. The reaction without AcNCSs was used as the negative control. The reactions of Δ19TfNCS from *T. flavum* and NnNCS1 from *N. nucifera* were used as positive controls. (**c**) MS/MS spectra of norcoclaurine and the products formed under the catalysis of AcNCS.

In order to further investigate the catalytic activity of the identified AcNCSs, five AcNCSs, including AcNCS2, AcNCS4, AcNCS5, AcNCS14, and AcNCS15, were selected based on the patterns of gene expression and norcoclaurine accumulation. The coding sequences of the *AcNCS* genes were cloned by PCR amplification and then ligated into the pET-30a vector. Expression of the AcNCS proteins was carried out using an *E. coli* expression system. After induction and purification, an in vitro catalytic activity assay of the recombinant AcNCS proteins was assessed using dopamine and 4-HPAA as substrates (Figure 7a). Δ19TfNCS from *T. flavum* and NnNCS1 from *N. nucifera* were used as positive controls. UPLC analysis shows that new peaks at a retention time of 5.4 min were generated under the catalysis of AcNCS2, AcNCS4, AcNCS5, AcNCS14, AcNCS15, Δ19TfNCS, and NnNCS1 recombinant proteins (Figure 7b). Their retention time is the same as that of the reference standard (*S*,*R*)-norcoclaurine (Figure 7b). Mass spectrum analysis shows that the new products and (*S*,*R*)-norcoclaurine had *m*/*z* values of 272 (Figure 7c). MS/MS spectrum analysis shows that the new products with *m*/*z* values of 272 were fragmented into main ions identical to those of the reference standard (*S*,*R*)-norcoclaurine (Figure 7c). The results suggest that the AcNCS proteins analyzed could catalyze the condensation of dopamine and 4-HPAA to norcoclaurine.

### 2.7. Affinity of AcNCSs Toward Dopamine and 4-HPPA

In order to characterize the detailed kinetics of AcNCS catalysis and investigate the differences in catalytic efficiency among the five AcNCSs, the affinities of the AcNCSs toward dopamine and 4-HPPA were analyzed. To test the affinities of the AcNCSs toward dopamine, AcNCS proteins were incubated with different concentrations of dopamine while keeping 4-HPPA saturate. The results show that the kinetic parameters of the different recombinant AcNCS proteins varied (Table 2). The *K*m values varied from 420.8 µM for AcNCS14 to 534.9 µM for AcNCS2. The *V*max values varied from 1.6 µM min^−1^ for AcNCS2 to 2.3 µM min^−1^ for AcNCS14 and 2.2 µM min^−1^ for AcNCS15. The *K*cat/*K*m values varied from 3.9 × 10^4^ M^−1^ S^−1^ for AcNCS2 to 4.3 × 10^4^ M^−1^ S^−1^ for AcNCS15 and 5.3 × 10^4^ M^−1^ S^−1^ for AcNCS14 (Table 2). This suggests that AcNCS14 and AcNCS15 have relatively high affinities toward dopamine, whereas the affinities of AcNCS2, AcNCS4, and AcNCS5 toward dopamine are relatively low.

To assess the affinities of the AcNCSs toward 4-HPPA, dopamine was kept saturate, whereas the concentration levels of 4-HPPA varied. The results show that the *K*m values of the AcNCSs for 4-HPPA varied from 495.3 to 536.4 µM. The *V*max values varied from 1.30 to 1.5 µM min^−1^. The *K*cat/*K*m values varied from 2.5 × 10^4^ to 2.9 × 10^4^ M^−1^ S^−1^ (Table 3). This suggests that AcNCS14 and AcNCS15 have relatively high affinities toward 4-HPPA, whereas the affinities of the other AcNCSs toward 4-HPPA are relatively low. The results are similar to the affinities of the AcNCSs toward dopamine (Table 2).

### 2.8. Existence of (S)- and (R)-Norcoclaurine in AcNCS-Catalyzed Products

**Figure 8:**
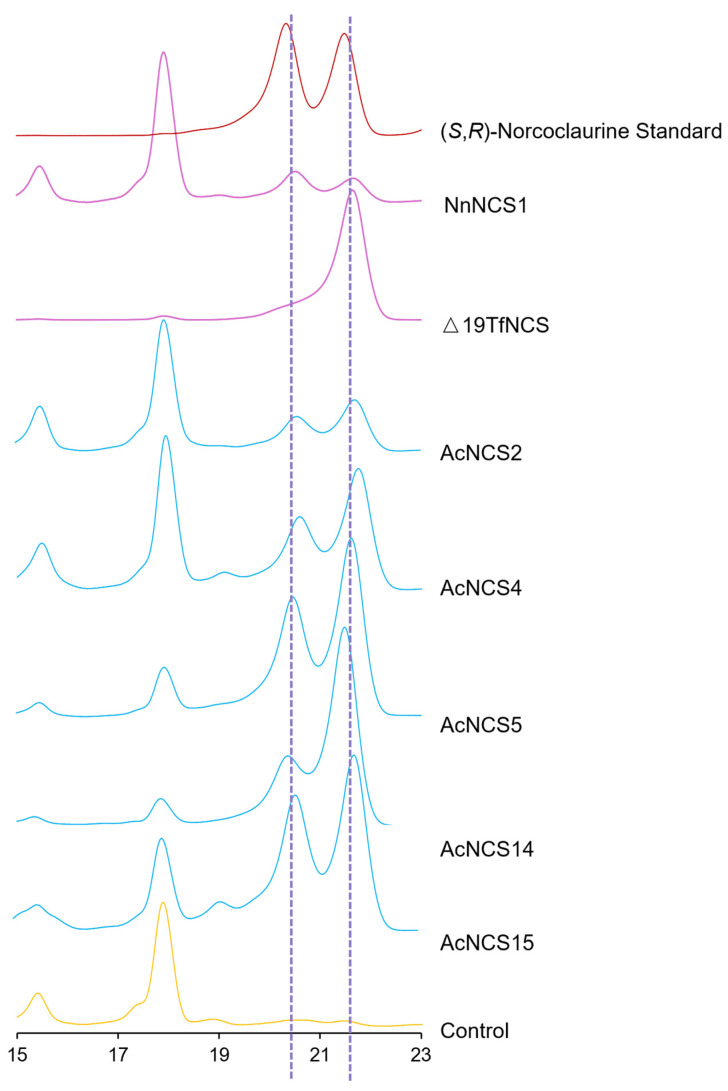
Configuration assays of norcoclaurine using precolumn derivatization. The (R, S)-norcoclaurine standard is shown in red. The products from the catalysis of the AcNCSs are shown in blue. The products from the catalysis of NnNCS1 and TfNCS were used as positive controls (pink). The reaction without AcNCS catalysis was used as the negative control (yellow).

Chirality could significantly affect the pharmacological activity of BIA alkaloids. NCSs are the first enzymes determining the chirality of BIAs. Previous studies show that NCSs from Ranunculales plants catalyze the generation of (*S*)-norcoclaurine, whereas those from *N. nucifera* catalyzed the production of both (*S*)- and (*R*)-norcoclaurine [24,25,27,42,43,44]. In addition, the reaction catalyzed by a gymnosperm *G. montanum* NCS generated the majority of products as (*S*)-norcoclaurine and a minor amount as (*R*)-norcoclaurine [44]. In order to elucidate the stereochemical characteristics of AcNCS products, we implemented a precolumn derivatization strategy through the application of R-(−)-DBD-PyNCS, as previously described [44]. NnNCS1 from *N. nucifera* and △19TfNCS from *T. flavum* were synthesized and used as positive controls. The results show that TfNCS specifically catalyzed the production of (*S*)-norcoclaurine, whereas NnNCS1 catalyzed the generation of an almost equal amount of (*S*)- and (*R*)-norcoclaurine (Figure 8), which is consistent with previous studies [44]. All of the AcNCS-catalyzed products exhibited two distinct enantiomers, with (*S*)-norcoclaurine being more abundant and (*R*)-norcoclaurine being less (Figure 8). The difference between the amounts of (*S*)- and (*R*)-norcoclaurine varied among the AcNCSs. The products generated under the catalysis of AcNCS14 showed the most significant difference (Figure 8). The results suggest that all five AcNCSs, including AcNCS2, AcNCS4, AcNCS5, AcNCS14, and AcNCS15, catalyze the production of (*S*)- and (*R*)-norcoclaurine, with (*S*)-norcoclaurine being more abundant than (*R*)-norcoclaurine.

## 3. Discussion

NCSs are important enzymes catalyzing the first committed step in the biosynthesis of BIAs and their derivatives in plants [21]. Since the first report of an *NCS* gene from *T. flavum*, various *NCS* genes have been identified and characterized from the order Ranunculales [22,25,26,27,28]. In addition, five NCSs from *N. nucifera* that belong to the order Proteales and an NCS from the gymnosperm *G. montanum* were also functionally analyzed [44]. Both Ranunculales and Proteales are positioned at the basal nodes of eudicots, whereas *A. contorta* belongs to a relatively basal node of angiosperms, although the taxonomic status of Magnoliids that *A. contorta* belongs to has long been debated [11,24,25,27,42,43,47]. From an evolutionary perspective, the identification and characterization of the 15 *AcNCS* genes is significant for elucidating the evolution history of plant *NCS* genes.

The phylogenetic analysis shows that AcNCS1, AcNCS2, and AcNCS8–AcNCS15 were included in clade NCS III (distinct from Ranunculales NCSs), whereas AcNCS3–AcNCS7 were included in clade NCS II (Figure 2). Catalytic activity analysis shows that AcNCS2, AcNCS14, and AcNCS15 from clade NCS III and AcNCS4 and AcNCS5 from clade NCS II could catalyze the condensation of dopamine and 4-HPPA to form norcoclaurine (Figure 7a). Taken together with previous results, this shows that NnNCS1, NnNCS3, NnNCS5, and NnNCS7 from clade NCS I and NnNCS4 from clade NCS II could catalyze the reaction [44]; it is possible that members of all three NCS clades have the potential to catalyze norcoclaurine formation. Considering that the catalytic activity and substrate affinities of the NCSs were not high, the evolution of multiple copies of the *NCS* genes could be important for providing sufficient substrates for subsequent reactions that lead to the biosynthesis of highly cumulative magnoflorine and AAs in *A. contorta* [11,44,46,48,49].

It has been shown that the Pictet–Spengler reaction can proceed spontaneously in the absence of enzymes [50]. However, the conditions required for such a spontaneous reaction are highly stringent for plants. So far, no evidence has been reported regarding whether this reaction occurs spontaneously in vivo in plants. In order to provide sufficient substrates for subsequent reactions, as mentioned above, BIA-producing plants evolve multiple copies of *NCSs*. On the other hand, some of the NCS enzymes evolved to possess tandem repeat structures. For instance, *P. somniferum* NCS has one to four tandem repeats [48]. Among the 15 AcNCSs identified in this study, 8, including AcNCS1–AcNCS8, had one catalytic domain. The other seven, including AcNCS9–AcNCS15, had two catalytic domains. Interestingly, an enzyme catalytic activity assay shows that AcNCS14 and AcNCS15, which have two catalytic domains, exhibit higher catalytic efficiency than AcNCS2, AcNCS4, and AcNCS5, which have a single catalytic domain. This indicates that increasing the number of catalytic domains could enhance the catalytic efficiency of AcNCSs.

NCS enzymes are stereoselective. Ranunculales NCSs specifically catalyze the generation of (*S*)-norcoclaurine [24,25,27,42,43]. NCSs from *N. nucifera* of Proteales catalyze the production of almost equal amounts of (*S*)- and (*R*)-norcoclaurine [44]. A gymnosperm NCS from *G. montanum* catalyzes norcoclaurine generation with the majority as (*S*)-configuration and a minority as (*R*)-configuration [44]. Precolumn derivatization analysis of the AcNCS-catalyzed products show that there were more (*S*)-norcoclaurine than (*R*)-norcoclaurine (Figure 8). Variation in the (*S*)- and (*R*)-norcoclaurine proportions could be due to changes in the amino acid residues of the NCS proteins. The key amino acid residues responsible for steroselectivity remain to be identified. In addition, the steroselectivity of NCSs seems to be associated with the substrate specificity of downstream enzymes in the BIA biosynthetic pathway. For instance, CjCYP80G2 from *C. japonica* exclusively accepts (*S*)-configured substrates, whereas NnCYP80Q1 from *N. nucifera* only accepts (*R*)-configured substrates [51,52]. AcCYP80G7 and AcCYP80Q8 from *A. contorta* lack stereoselectivity; they can accept (*S*)- and (*R*)-configured substrates with equal efficiency [12]. The STORR fusion enzyme in the BIA pathway of Ranunculales can convert (*S*)-reticuline to (*R*)-reticuline [37,38]. These results highlight the diversity of stereochemical conversions within the BIA pathway. Currently, the chirality of BIA in *A. contorta* and the mechanisms underlying stereochemical conversion in *A. contorta* remain largely unknown, which requires further investigation.

## 4. Materials and Methods

### 4.1. Plant Materials and Chemicals

The roots, stems, leaves, and flowers of *A. contorta* Bunge used for the gene expression profiling and norcoclaurine content determination were collected from the Medicinal Botanical Garden at the Institute of Medicinal Plant Development, Beijing, China. After labeling, the collected plant samples were rapidly frozen in liquid nitrogen and stored at −80 °C until use. The reference standards, including dopamine, 4-hydroxyphenylacetaldehyde (4-HPAA), and (*S*, *R*)-norcoclaurine, were purchased from Beijing Xinhuitian Dongfang Biotechnology (Beijing, China), Toronto Research Chemicals (Toronto, ON, Canada), and Chendu Biopurify Phytochemicals (Chengdu, China), respectively.

### 4.2. AcNCS Gene Identification

The amino acid sequences of the four published NCSs, including TfNCS (ACO90248.1), PsNCS1 (AAX56303.1), PsNCS2 (AAX56304.1), and CjNCS2/CjPR10A (A2A1A1.2), were downloaded from the National Center for Biotechnology Information’s (NCBI) website at https://www.ncbi.nlm.nih.gov accessed on 25 July 2023. Blast analysis of the five proteins against the *A. contorta* whole genome assembly was carried out using the tBLASTn algorithm with an *e*-value cut-off of 1 × 10^−5^ [11,53]. *AcNCS* gene models were predicted from the retrieved genomic DNA sequences through alignment with *NCS* genes from other plant species using the BLASTx algorithm with the default parameters (https://blast.ncbi.nlm.nih.gov/Blast.cgi accessed on 28 July 2023). The predicted gene models were further compared with the protein-coding genes annotated for the whole genome assembly of *A. contorta* through BLASTn analysis [11,53].

### 4.3. Analyses of Sequence Features, Chromosomal Locations, and Gene Structures

The amino acid compositions, molecular weights, and theoretical isoelectric points were determined using the ProtParam tool (https://web.expasy.org/protparam/ accessed on 13 September 2023). Chromosomal locations of the *AcNCS* genes were analyzed by comparison analysis of the gene sequences with the whole genome assembly of *A. contorta* and visualized using TBtools v2.210 [11,54]. Gene structures were determined through comparison of the coding sequence with the whole genome assembly of *A. contorta* and visualized using TBtools [11,54]. Conservative domains and conservative motifs were analyzed on the Batch-CD (https://www.ncbi.nlm.nih.gov/Structure/bwrpsb/bwrpsb.cgi) and MEME (https://meme-suite.org/meme/ accessed on 5 December 2023) websites, respectively.

### 4.4. Phylogenetic Tree Construction

Amino acid sequences of 55 NCSs and 11 PR10 proteins from various plants were downloaded from NCBI (Tables S2 and S3). Sequence alignment was performed using ClustalW in MEGA 11 with the default parameters [45]. The neighbor-joining method was utilized to conduct a phylogenetic analysis with a bootstrap value of 1000 replicates [45].

### 4.5. Quantitative Real-Time Reverse Transcription-PCR (qRT-PCR)

The total RNA was extracted from the roots, stems, leaves, and flowers of *A. contorta* using the Quick RNA Isolation kit (Huayueyang, Beijing, China). The quality of the extracted RNA was assessed by electrophoresis with 1.2% agarose gel. The concentration of the extracted RNA was determined using a Nanodrop2000 (ThermoFisher Scientific, Waltham, MA, USA). cDNAs were synthesized from 1 μg of total RNA using the TRUEscript 1st Strand cDNA Synthesis kit (Aidlab, Beijing, China) as per the manufacturer’s instructions. Gene expression was determined by qRT-PCR using SYBR Green qPCR Mix (Aidlab, Beijing, China). β-Actin was utilized as the internal reference gene. Three biological and three technical replicates were carried out. Gene-specific primers were designed on the IDT server (https://sg.idtdna.com/scitools/Applications/RealTimePCR/Default.aspx accessed on 10 August 2024). The primers are listed in Table S5. The thermal profile for the qRT-PCR was as follows: pre-denaturation at 95 °C for 3 min, followed by 40 cycles of 95 °C for 15 s, 60 °C for 20 s, and 72 °C for 30 s. Melting curves were obtained by treating the PCR products at 95 °C for 5 s, 65 °C for 5 s, and 95 °C for 15 s. The gene expression levels of the AcNCS genes were calculated using the 2^−ΔΔC^**^t^** method after normalization to the internal reference gene.

### 4.6. Norcoclaurine Content Determination

The contents of norcoclaurine in the roots, stems, leaves, and flowers were determined using LC-MS/MS. The roots, stems, leaves, and flowers were dried for 24 h in an oven at 37 °C (DH5000II, TAISTE, Tianjing, China). Norcoclaurine was extracted from 0.2 g of roots, stems, leaves, and flowers by ultrasonic extraction with 80% methanol. The LC-MS/MS system, which consisted of an Agilent 1260 Infinity HPLC system (Agilent Technologies, Santa Clara, CA, USA) equipped with an Agilent Poroshell 120 SB C18 column (100 mm × 2.1 mm, 2.7 µm) and an AB SCIEX 4500 QTRAP MS system (AB SCIEX, Redwood City, CA, USA), was used for the sample analysis. The mobile phase for the gradient elution consisted of (A) water (containing 0.1% formic acid) and (B) acetonitrile with the following gradient procedure: 0–0.3 min, 90% A; 0.3–3 min, 90–2% A; 3–3.5 min, 2% A; 3.5–4 min, 2–90% A; and 4–5 min, 90% A, with a flow rate of 0.25 mL/min. The injection volume was 5.0 µL. Electrospray ionization (ESI) in the positive-ion mode and multiple reaction monitoring (MRM) scanning was employed for the quantification. The curtain gas (CUR), nebulizer gas (GS1), and auxiliary gas (GS2) were set at 15, 40, and 50 psi, respectively. The ion spray voltage (IS) was adjusted to 4000 V for the positive-ion mode, and the source temperature was maintained at 350 °C. Chromatographic workstation (v1.6.2) was used for the data analysis.

### 4.7. AcNCS Recombinant Protein Expression and Purification

Full-length coding sequences of the *AcNCS* genes were amplified by PCR using Phanta Flash Master Mix (Vazyme, Nanjing, China). The primers used for cloning are listed in Table S6. The vector pET-30a was linearized by the endonucleases *Nde*I and *Not*I. PCR products were subcloned into a pET-30a vector through homologous recombination using the ClonExpress II One-Step Cloning kit (Vazyme, Nanjing, China). The C-terminal of the recombinant protein carries a His label. The cloned sequence was confirmed by sequencing. The vector with a correct sequence was introduced into *E. coli* strain BL21 (DE3). Cells were incubated at 37 °C in Luria–Bertani (LB) medium until reaching an OD600 of 0.6–0.8. The AcNCS recombinant proteins were induced with 0.5 mmol L-1 IPTG at 16 °C for 20 h. The cells were collected by centrifugation for 10 min at 8000 rpm in 4 °C and then resuspended in 20 mM HEPES buffer I (500 mM of NaCl and 20 mM of imidazole, pH 7.4). After harvesting, the cells were sonicated on ice. Crude AcNCS proteins were purified using a Ni-NTA column (Abbkine, Wuhan, China) with buffer II (20 mM of HEPES, 500 mM of NaCl, and 300 mM of imidazole, pH 7.4). Purified proteins were concentrated and desalted in 20 mM HEPES buffer III (500 mM of NaCl, 20 mM of imidazole, and 10% glycerol, pH 7.4) using an Amicon Ultra-15 mL Centrifugal Filter (Millipore, Burlington, MA, USA). Aliquots were stored at −80 °C. The purity of the recombinant proteins was examined by SDS-PAGE. The protein concentration was quantified using the BCA Protein Assay kit (Takara, Beijing, China).

### 4.8. In Vitro Enzymatic Activity Assay of AcNCS Recombinant Proteins

The reaction was carried out in a 100 μL reaction system consisting of 50 mM HEPES buffer (pH 7.4). The reaction system contained 2 mM of dopamine, 2 mM of 4-HPAA, 1 mM of ascorbic acid, and 50 μg of proteins. The reaction was conducted at 42 °C for 30 min and then terminated by the addition of 50 μL methanol. The mixture was centrifuged at 13,000 rpm for 10 min, and the supernatant was used for the assay. Reaction products were separated on an ACQUITY UPLC BEH C18 column (1.7 μm, 2.1 × 100 mm) using an ACQUITY UPLC system (Waters, Milford, MA, USA). Samples (2 μL) were eluted with 0.1% formic acid in carbinol (A) and 0.1% formic acid in water (B) based on the following gradient: 0–1 min, 3% A; 1–11 min, 3–97% A; 11–16 min, 97–3% A; and 16–20 min, 3% A. The flow rate was 0.3 mL/min, and the photodiode array (PDA) spectrum was 280 nm. Mass spectrometry detection was performed on an Orbitrap Exploris 120 (ThermoFisher Scientific) mass spectrometer coupled with an Xcalibur in the positive-ion mode based on the information-dependent acquisition mode.

### 4.9. Kinetic Analysis of AcNCS Recombinant Proteins

The kinetics of the AcNCS recombinant proteins were analyzed in a 100 μL reaction system consisting of 50 mM of HEPES buffer, 50 µg of recombinant proteins, and different concentrations of the substrate. The reaction was carried out at 42 °C for 30 min, and then 100 µL of methanol was added to terminate the reaction. The reaction products were analyzed using a UPLC system, as described in Section 4.8. Enzyme activities were determined by measuring the changes in the substrate content. To determine the kinetic parameters, the saturation concentration of one substrate was set to 2 mM, while the concentration of the other substrate was varied at different levels, including 10, 100, 200, 500, 1000, 2500, and 5000 µM. The kinetic constant of the substrate was determined by monitoring the consumption of the substrate. The enzyme assay was repeated three times at each substrate concentration. The *V*max and *K*m values were calculated using nonlinear regression analysis with Graph Prism 9.0 software.

### 4.10. Chiral Analysis of the Configuration of Products

Enzymatic reaction products were extracted with ethyl acetate. Then, the ethyl acetate was evaporated using a nitrogen-blowing instrument. The resulting precipitate was resuspended in 50 μL acetonitrile. An equal volume of 3% pyridine acetonitrile solution and 30 mM R-(-)-DBD-PyNCS acetonitrile were added. After mixing thoroughly, the reaction was allowed to proceed at 60 °C for 1h. The reaction products were detected using an Agilent 1260 liquid chromatography system (Agilent Technologies, Santa Clara, CA, USA) equipped with an Agilent Zorbax Eclipse Plus C 18 column (250 × 4.6 mm, 5 µm). The mobile phase consisted of 0.2% formic acid in water–methanol–acetonitrile (45:30:35, *v*/*v*/*v*) at a flow rate of 0.5 mL/min. The total running time was 35 min, injection volume was 10 μL, flow rate was 0.5 mL/min, and column temperature was 35 °C. Detection was performed using a UV detector at a wavelength of 280 nm.

## 5. Conclusions

Through genome-wide identification, a total of 15 *A. contorta NCS*s were identified. The conserved domain analysis showed that eight of them contained one catalytic domain, whereas the other seven had two. The phylogenetic analysis showed that 15 AcNCS proteins can be classified into two clades. The gene expression analysis showed that five *AcNCSs* exhibited relatively high expression in tissues where norcoclaurine accumulated. The enzyme catalytic activity assay and precolumn derivatization analysis showed that all of the five AcNCSs could catalyze the formation of (*S*)- and (*R*)-norcoclaurine, revealing the uniqueness and complexity of the BIA pathway in *A. contorta*. This is the first report of a functional analysis of AcNCSs. The results provide useful information for manipulating the production of BIAs and their derivatives in *A. contorta* through genetic and biotechnological approaches and serve as a foundation for the development of low-toxicity or non-toxic medicinal plant resources. Future studies could concentrate on exploring the mechanisms of the stereochemical conversion of BIAs and their derivatives in *A. contorta*. The downstream pathway leading to AA biosynthesis also requires intensive study.

## Figures and Tables

**Table 1:** Features of *AcNCS* genes and their deduced proteins.

Gene Name	Sequence ID	Number of Amino Acids	Molecular Weight (kDa)	Theoretical Isoelectric Point (pI)	Number of Introns	Chromosome Localization
*AcNCS1*	EVM0011227.1	160	17.6	5.12	1	LG02
*AcNCS2*	EVM0014712.1	160	17.6	4.99	1	LG02
*AcNCS3*	EVM0011942.1	164	18.1	5.05	1	LG03
*AcNCS4*	EVM0001864.1	164	17.9	4.91	1	LG02
*AcNCS5*	EVM0010147.1	164	17.9	5.01	1	LG02
*AcNCS6*	EVM0012460.1	173	18.6	5.05	1	LG02
*AcNCS7*	EVM0000189.1	173	18.6	5.05	1	LG02
*AcNCS8*	EVM0011298.1	194	21.3	5.24	1	LG01
*AcNCS9*	EVM0016647.1	303	33.8	5.69	2	LG02
*AcNCS10*	EVM0005050.1	313	34.6	5.00	2	LG02
*AcNCS11*	EVM0007329.1	318	35.5	6.15	1	LG02
*AcNCS12*	EVM0000274.1	318	35.5	6.15	1	LG02
*AcNCS13*	EVM0013491.1	323	35.5	4.63	2	LG01
*AcNCS14*	EVM0002685.1	355	39.0	5.40	2	LG02
*AcNCS15*	EVM0014575.1	355	39.0	5.39	2	LG02

**Table 2:** Kinetic parameters of the recombinant AcNCS proteins toward dopamine.

Protein	*V*max (µM min^−1^)	*K*m (µM)	*K*cat (S^−1^)	*K*cat/*K*m (M^−1^ S^−1^)
AcNCS2	1.6 ± 0.1	518.9 ± 109.9	20.3 ± 1.0	3.9× 10^4^
AcNCS4	1.6 ± 0.2	531.5 ± 86.5	21.4 ± 2.3	4.0 × 10^4^
AcNCS5	1.6 ± 0.2	534.9 ± 78.3	21.3 ± 2.1	4.0 × 10^4^
AcNCS14	2.3 ± 0.2	420.8 ± 45.9	22.1 ± 1.3	5.3 × 10^4^
AcNCS15	2.2 ± 0.2	502.9 ± 114.6	21.8 ± 1.4	4.3 × 10^4^

**Table 3:** Kinetic parameters of the recombinant AcNCS proteins toward 4-HPAA.

Protein	*V*max (µM min^−1^)	*K*m (µM)	*K*cat (S^−1^)	*K*cat/*K*m (M^−1^ S^−1^)
AcNCS2	1.3 ± 0.2	522.5 ± 72.6	13.1 ± 1.2	2.5 × 10^4^
AcNCS4	1.3 ± 0.2	530.3 ± 76.4	13.2 ± 2.0	2.5 × 10^4^
AcNCS5	1.4 ± 0.2	536.4 ± 78.6	13.2 ± 1.9	2.5 × 10^4^
AcNCS14	1.5 ± 0.2	495.3 ± 98.2	14.6 ± 1.2	2.9 × 10^4^
AcNCS15	1.5 ± 0.2	506.7 ± 63.8	14.2 ± 1.4	2.8 × 10^4^

## Data Availability

The data are available in the article and its Supplementary Materials.

## Supplementary Materials

The following supporting information can be downloaded at: https://www.mdpi.com/article/10.3390/ijms26094314/s1.
