# Supplementary material for: Genome-Wide Identification and Functional Analysis of the Norcoclaurine Synthase Gene Family in Aristolochia contorta
Source: Int J Mol Sci. 2025 May 1;26(9):4314. doi: 10.3390/ijms26094314 (PMC12072525; doi:10.3390/ijms26094314)
Supplement: Supplementary file 1 [file ijms-26-04314-s001.zip › ijms-3570354-supplementary.pdf]

International Journal of *Molecular Sciences*

MDPI

Table S1. Sequences of *AcNCSs*

Gene name	Coding sequence
AcNCS1	ATGCGGGGGAAGAAGATCAGCGAAATCGACGTAGCTTTCCCCGCTTCTGTCGTGTGGGATGTGTACAGCA<br>CGACGAAGCTATCGGAAGTCGTAAAAACAATCCCCAATTTCCGAGGGAAGCAGCAGGTCATCGGCGACG<br>GCGGCGTCGGAACCATCATCTCCGGCGAACTACCACCGGGAGGTCCTTACGGAGCGTATAAGGAGAAGT<br>GGGTGACGGTGGATCATGAGAACCGGGTGAAGGAAACGGACACCATCGAAGGCGGAGTTCTCGACGTGG<br>GTTTCGTTTTCTACCGGCTTCGGTTTGAAATCGTGGAGGAGGGTTCGAATGCCTCGAGGATCAAAACCATA<br>ATCATGTACGAGATTAAAGACGAGTTCGCAGCCGCGAATGAATATATAGCAGATCAGAACCTTGCACAG<br>ATGGAGACGATGGCTAGAGCTGTGGCCAAGTATCTGGCTAATATAAACGCCGTCGCCCCAAGCTAA
AcNCS2	ATGCGGGGGAAGAAGATCAGCGAAATCGACGTAGCTTTCCCCGCTTCTGTCGTGTGGGATGTGTACAGCA<br>CGACGAAGCTATCGGAAGTCGTAAAAACAATCCCCAATTTCCGAGGGAAGCAGCAGGTCATCGGCGACG<br>GCGGCGTCGGAACCATCATCTCCGGCGAACTACCACCGGGAGGTCCTTACGGAGCGTATAAGGAGAAGT<br>GGGTGACGGTGGATCATGAGAACCGGGTGAAGGAGACGGACACCATCGAAGGCGGAGTTCTCGACGTGG<br>GTTTCGTTTTCTACCGGCTTCGGTTTGAAATCGTGGAGGAGGGTCCGAATGCCTCGAGGATCAAAACCATA<br>ATCATGTACGAGATTAAAGACGAGTTCGCAGCCGCGAATGAATATATAGCAGATCAGAACCTTGCACAG<br>ATGGAGACGATGGCTAGAGCTGTGGCCAAGTATCTCGCTGATATAAACGCCGCCGCCCCAAGCTAA
AcNCS3	ATGCACGGCCATCTCGCCAGCGATTTGGAACTCCGGGCACCGGCGGCGAGGGTGTGGGAATGCTACAGC<br>ACCCTCGAGCTCCACCACGTCGTCAGCCATTACCTCGCCCAATTCGTCGAGAAGATTGACATCGTTCAAG<br>GCGACGGAAAAGTCGGCACCATTCTCGACATCTTTTTCGTGGAAGGTACGCCGGGAGTTCGTCGCTACAA<br>GGAGAAGTTCATCGTGGTGGACGACGCGACGAGGGTGAAGACCGCCGACGCCATTGAAGGCGGCTTCCA<br>AGAACTGGGTTTCGGACTCTACCGCGTCTCGTTTCAGATTGTGGACAAGGAACCCGGTACTTCCTGCATCG<br>TCAAAGCCACTATCGACTACGAGATCGACGACGACAAGGTCTCCAATGCGTCCTTCGTCAGCACCGCCGT<br>GCTGGATGTTATGGGGAAAGCGGTGGAGCAGCACATCATCGGTCAAAACAAACCGGAGGCTGCTCAACC<br>AAACTAA
AcNCS4	ATGCACGGTCAGGTCTCGCACGAGCTCGAGGTAAAGCAACCGGCGAGGAAGGTATGGGAAATCTACGGA<br>ACGCTTAAGCTGGGGAAACTCGTCGTGCAGAAGCTCCCCAACATCATCGAAAAGATCGACATAGACGGT<br>GATGGAGGAGTCGGCACTGTTCTCACCGTCTTTTTCCCAAACGGGTCTGGTAGGTTCAAGGAGAAGTTCGT<br>GGTGATCGACGATGAGAAGAGAGTGAAGGTTACGGAAGTGGTGGAAGGTGGGCTTCTCGATCTGGGTTTC<br>GCCTCAAACCGTACTACTCTGGAGATCTTAGAGAAAGATGATCAAGGACGAAATTCGTGCATCATCAGGT<br>CGACCATTGACTATGAGATTGGGGATGAAAACGCCTCCTCTAAGTCTTCGCTCGTCACCACGGCTGCACTT<br>GAGGTGATTGCGCAGATGGTTGCACAGCATCTCGGTGAAATTAGCAATGGGGAGATCGGATATGCAGAC<br>GCTTGA
AcNCS5	ATGCACGGTCAGGTCTCGCACGAGCTCGAGGTAAAGCAACCGGCGAGGAAGGTATGGGAAATCTACGGA<br>ACGCTTAAGCTGGGGAAACTCGTCGTGCAGAAGCTCCCCAACATCATCGAAAAGATCGACATACACGGT<br>GATGGAGGAGTCGGCACTGTTCTCACCGTCTTTTTCCCAAACGGGTCTGGTAGGTTCAAGGAGAAGTTCGT<br>GGTGATCGACGATGAGAAGAGAGTGAAGGTTACGGAAGTGGTGGAAGGTGGGCTTCTCGATCTGGGTTTC<br>GCCTCAAACCGTACTACTCTGGAGATCTTAGAGAAAGATGATCAAGGACGAGATTTGTGCATCATCAGGT<br>CGACCATTGACTATGAGATTGGGGATGAAAACGCCTCCTCTAAGTCTTCGCTCGTCACCACGGCTGCACTT<br>GAGGTGATTGCGCAGATGGTTGCACAGCATCTCGCTGAAATTAGCAATGGGGAGATCGGATATGCAGAC<br>GCTTGA
AcNCS6	ATGCAGGGTCGGGTCTCGCATGAGCTCGAGGTGGAACAACCGGCGAGCAAGGTATGGGAAATCTACGGG<br>TCACTCAAGCTGGGTAAACTCATCATGCAGAAGCTTCCCCACATGATCGAGAAGATTGACATAGACGGCG<br>ATGGAGGAGCCGGCACTGTTCTCACCGTCACTTTCCCCAAAGGCACGCGAGCGTTTAGTTCGTTCAAGGA<br>GAATTTTGCAGTGATCGACAATGAGAAGAGAGTGAAGGTAGCGGAAGTGGTGGAAGGTGGGTTTCTGGA<br>CGTGGGTTTCGCCTCACACCGTACTACTCTGGAGATCGCTGCGGAGAAAGAATTAGGTGGTAGCAGTTCG<br>ACGTGCATCATCAGATCGACCATTGAGTATGAAATTCGCGACGAAGAGGCCTCCAAATGTTCGCCCGTCA<br>CCATTGCCGGAATTGAGCTGGTAGCAAACATGGTCGCGGACCATCTCGCAGAGATCGACGACGGGAAGT<br>CTAAGAATGTGGGATCTGGATCAGATCACGCTTGA
AcNCS7	ATGCAGGGTCGGGTCTCGCATGAGCTCGAGGTGGAACAACCGGCGAGCAAGGTATGGGAAATCTACGGG<br>TCACTCAAGCTGGGTAAACTCATCATGCAGAAGCTTCCCCACATGATCGAGAAGATTGACATAGACGGCG<br>ATGGAGGAGCCGGCACTGTTCTCACCGTCACTTTCCCCAAAGGCACGCGAGCGTTTAGTTCGTTCAAGGA<br>GAATTTTGCAGTGATCGACAATGAGAAGAGAGTGAAGGTAGCGGAAGTGGTGGAAGGTGGGTTTCTGGA<br>CGTGGGTTTCGCCTCACACCGTACTACTCTGGAGATCGCTGCGGAGAAAGAATTAGGTGGTAGCAGTTCG<br>ACGTGCATCATCAGATCGACCATTGAGTATGAAATTCGCGACGAAGAGGCCTCCAAATGTTCGCCCGTCA

 https://doi.org/10.3390/10.3390/ijms26094314

	CCATTGCCGGAATTGAGCTGGTAGCAAACATGGTCGCGGACCATCTCGCAGAGATCGACGACGGGAAGT<br>CTAAGAATGTGGGATCTGGATCAGATCACGCTTGA
AcNCS8	ATGGCCACTGTTACGAAGGGCATTTTGCACGAAACAAAAAATGAAGTCGCTGTTAAAGAGTCTCTGGCCA<br>CTGTAGAAGAAAACTTTCTTACCAGCAGGAGCGCCAGTACTGCTGGTCTCATACTTGGGGAACAGATAAT<br>CTCAGTGGATATACACTTCTCCGCACAAGAGGTATGGGACGTCTTCGCCAACACGCACCAGCTTGCTCAA<br>ATCTTCAAGACCGTGCCTAGTTCTGGCTTCGAGGACATTCAGGTTGTTGGTGATGGAGGTCTCGGAACCCT<br>CATCATCCTCATCCTCAGACCAGGGAGCACTTTCAGTCAATTCACGGAGACCTTGGTGACATTGGACCATG<br>AACGAAGAGTGAAGGAAGTGGATGTGAGCGAAGGAGGATATCTGACTGTGGGCTACACGTTCTACCGCA<br>CTCGATTCGAGATCCTGCCCAAGGGTCCAAACAGGGCGACTGTCCGAAAGAGCATTATCTACAATGTGAG<br>AGAAGACTCAGCTGCTAACCTCTCTCTTGCCTCTGCTAATGACAATCCAGATCAAGTTAAAGCCGTGGAG<br>AACTTCCTAAGCAGGCAGCTCTAG
AcNCS9	ATGTGGGAAGTAGTAAGCACACTCGAGCTTCCGCAACTCGTCAAGAACATGCCCGGCGTCCTCAAAGATC<br>TCACCGTCGTCGGCGACGGATCGGTCGGCACCATCTTCAACCTCGTCGTCCCCGACGGGTGGCCATTTCAG<br>ACGTACAGAGAGAGGATCGCGACGCTGGACCACGCTAAGAGGCTCAAGGAAGTGAACGCGATCGAAGG<br>AGGAGTATTGAACTTGGGCTTCACCTTCTGTCAAACTCGATTCGAGGTTATCGACCAGGGTCCGACCACGT<br>CCATGTTCAGGGGCACCATCGTGTTCGAGATCGACGACACCAAAGCGACCCAGAAAGTTGTGGACATGGT<br>TGATCTCATGGCCATGGACTCCATTGCAAAGGCCATAGCGAATTACATAGTGGATAAAATATCTCACCCG<br>AGGAAGCTCGCCGGCGAGCAGTCCTTCGACCAGGACCTCCCTTACCCTGCAGCCGCCGTTTGGGATGTCT<br>ACAGCACGTTGAAGCTCCATGAACTATTCAGGAAGGTGGTTCCTGACGTCCAGTGGCAAGGAGATGGTTC<br>CGTTGGGACCATATTCTCATTCACCCTTAATCCAGGTTCCATTCCCAACTTCAAAGAAAGGCTCGTAAGAC<br>TGGACCAGAAGGAACGAATCAAGGAACTGAACGTGATCGAAGGAGGAGTTCTGGAAAAGGACTTCACC<br>GTCTACCGTACAAGGTTTCAGATCGTCGAAAACGGTCCAAACTCGACCGTGGTCAAGAACAGCATCATGT<br>ACGAACTGAATGCCAACTCCGCGACGGCGGCGGAGAACATGAAGCTCGCCAATGTTATGAAGCTCGCCA<br>ATGTTATGATAACGAGGACCGCCGGAGAACTGACCTCCGATTACTTGAAAAACCATAACAAATTAGGCG<br>CATGA
AcNCS10	ATGAAAGGCCAGCGTGTGCACGAATACGTCGTCCCCTTCCCTGCAGCCGATGTATGGGAAATTGTTGGCT<br>CCCTCGAGCTGCCGCAGCTCGTCAGAAACATGCCCGGAGTCCTCGAAGACCTTACCGTGATCGGCGACGG<br>CTCTGTTGGCACCATCTTCGTCCTCACCGTCCCCGACGGTTGGCCATTTAAGGTGTACAGGGAGAGGATCG<br>CGACGCTGGACCACGGTACGATGCTGAAGAACGTGGACGTGATCGAAGGCGCGGTTTTGGACTTTGGGTT<br>CACCTCCTGCACCACTCGGTTCGAGGTCTTCAAACTGGGGAGCTCCGTCTCCATGTTCAGGGGCACCATCC<br>TCTTCGAGATCGACGACGCCAAGGCGACCCAGACCGTCGTCGACATGATCGACCTCATGCAAATGGACG<br>GCGTCGCCAACGCCATCGCGCATAACATAGTCGACAAAAAATCTTACGCCAGGAAGCTCGCCGGCGACC<br>AGTCCTTCGAACGCGCCGTGTCCCACCCCGCCGCCGACGTTTGGGATGTCTACAGCACCCTCAAGCTCCCC<br>GAACTGTTAAAGCAAGTCTTCCCTGATGTTCAGTGGCAAGGAGACGGTTCCGTCGGGACCATTTACACTCT<br>CACCCTTAATCCCGGTCCGCTTCCGAGCTACAAAGAGAAGGTGGTGACGCTGAACCACGAGAAGCGAAT<br>CAAGGAACTCGACGTGATCGAGGGAGGGTACCTGGAGAAGGACTTCACCTCCTACCGCACAAGGTTCGA<br>AATCATCGAGACGAGCCCGACCTCGTGCATCATCAGGTCCATCATCATCGTCGAGCTGAACCAGAACGCC<br>ACTACGGCCGCCGAAAACATGAAGCTCGCCAATGTGATGGTGTTGAAGGCCGCCGGAGATGTCACCGCC<br>GATTACCTGACAAACAAAAGCCAAGCCGCATGA
AcNCS11	ATGAAAGGCCAGCGAGTGCACGAATACGTCGTCCCCTTCCCTCCACCCGATGTATGGGACGTCGTGGGCT<br>CGCTCCAGCTGCCGCAACTCGTCAACAACATGCCAGGGGTGCACCGTAATCTCACGGTGATGGGCGACGG<br>CTCCGTGGGCACCGTACTGATTTTCCTGTACGTTGCCGGATGGCCATTTAAGGGGTTCAGAGAGAGGATCT<br>CGTCGCTGGACCATGCGAACCGGGTCAAGGACGTCGACGTGATCCAAGGCGGGGTTCTCGACTACGGCTA<br>CACCTCCTGCCGCACCCGGTTCGAGGTCCTCGACCAGGGTTCCGCGGATCCGGAGAAGTACTCCAGCCTG<br>TTCAGGAGCACCGTCCTGTTCGAGATCGACGACGAGAAAGCGCCGGATAACGCCGTGGAGATGGTGGCG<br>CTCATGCAAATGGACGCCGTCGGGAAGGCCGTCGCGAATTACTTGGTGAATAAAAAGTCTCACCCGAGG<br>AAGCTCGCCGGCGAGCAGTCGTTCGACCAGGAGGTACCCTTCCCCGCCGCCGACGTTTGGAATGTCTACA<br>GCACACTGAAGCTCCCCGAACTGTTCAGAAAAGCGGTCCCTGATGTCCAGTGGCAAGGAGACGGCTCAG<br>TTGGAACCATATTCTCCGTCACCCTTCAACCAGGTCGTCTTCCAAACTACAAAGAGAGGCTCGTAACGCTG<br>GACCAGAAGGAACGGGTGAAGGAACTGGACGTTATCGAGGGAGGAGTTCGGGTAAGGGACTTCACCTTC<br>TACCGCATAAGGTCTCAGATCGTGGAAAACGGTCCAAACTCGACGGTGGTGAAGAATAGTATTAGATAC<br>GAACTGAATGCTAGCTCCCCGACGTCGGCAGAGAACATGAGGCTCGCCAATATTATGATTGTGAAAACAG<br>CCGGGGACCTCACCTCCGATTACCTCAAAACCATAAAGACAGGCGCATGA
AcNCS12	ATGAAAGGCCAGCGAGTGCACGAATACGTCGTCCCCTTCCCTCCACCCGATGTATGGGACGTCGTGGGCT<br>CGCTCCAGCTGCCGCAACTCGTAAACAACATGCCAGGGGTGCACCGTAATCTCACGGTGATGGGCGACG<br>GCTCCGTGGGCACCGTACTGATTTTCCTGTACGTTGCCGGATGGCCATTTAAGGGGTTCACAGAGAGGATC<br>TCGTCGCTGGACCATGCGAACCGGGTCAAGGACGTCGACGTGATCCAAGGCGGGGTTCTCGACTACGGCT<br>ACACCTCCTGCCGCACCCGGTTCGAGGTCCTCGACCAGGGTTCCGCGGCTACGGAGAAGTACTCCAGCCT

	GTTCAGGAGCACCGTCCTGTTCGAGATCGACGACGAGAAAGCGCCGGATAACGCCGTGGAGATGGTGGC
	GCTCATGCAAATGGACGCCGTCGGGAAGGCCGTCGCGAATTACTTGGTGAATAAAAAGTCTCACCCGAG
	GAAGCTCGCCGGCGAGCAGTCGTTCGACCAGGAGGTACCCTTCCCCGCCGCCGACGTTTGGAATGTCTAC
	AGCACACTGAAGCTCCCCGAACTGTTCAGAAAAGCGGTCCCTGATGTCCAGTGGCAAGGAGACGGCTCA
	GTTGGAACCATATTCTCCGTCACCCTTCAACCAGGTCGTCTTCCAAACTACAAAGAGAGGCTCGTAACGCT
	AGACCAGAAGGAACGGGTGAAGGAACTGGACGTTATCGAGGGAGGAGTTCGGGTAAGGGACTTCACCTT
	CTACCGCATAAGGTCTCAGATCGTGGAAAACGGTCCAAACTCGACGGTGGTGAAGAATAGTATTAGATAC
	GAACTGAATGCTAGCTCCCCGACGGCGGCAGAGAACATGAGGCTCGCCAATATTATGATTGTGAAAACA
	GCCGGGGACCTCACCTCCGATTACCTGAAAACCATAAGAGTAATGCTATGA
AcNCS13	ATGGTGGTGAGGGGAAAGCAAGTAAGCGAGGCTGACATTCCTTTCTCTGCCGCCGAGGTGTGGGGCGTGT
	ATGCAGACACGCATCAGCTGGCTCAAATCTTCAAGACAGTGCCGGGTTCTCTTGAGGACATCATAATTGA
	GGGCGATGGAGGTCTGGGGACCCTCATTATCCTCATCATCAAGCCAGGGAACGCTTTCAGGCAGTTTACA
	GAGAATGTCGTGACATTGAACAGCGAGGAGAGAGTAAAGGAAGTGGATGTGCAGGAAGGAGGGTTTCTT
	GACGTGGGATACACTTTCTACAGGACCCGTTTCGAGATCGTAGAAAAGGGTCCCAACATGGCCACTGTCA
	AGAAGAGTATTCTGTATGAAATCAACGACGAATTCGCTGCTAACGAGTCTCTGGCCACAGTTGAAGAAAA
	CCCAGCTCAAGTTAAAGCTGTTGAAAACTTTCTTACCAACAGGAGCAGCACTGATGGCCTCATACGAGGG
	GAACAGATAATCTCCGTGGACATACCATTCTCTGCACAACAGGTATGGGATGTCTACGCCAACACGAGTC
	AGCTTGCTCAAGTCTTCTTGACCGTTCCTGGGACTGCACTCGAGGACATTCAGGTTGTTGGTGATGGAGGT
	CTCGGAACCCTCATCATCCTCATCCTCAGACCAGGGAGCACTTTCAGTCAATTCACGGAGACCTTGATGAC
	ATTGGACCATGAACGAAGAGTGAAGGAAGTGGATGTGAGCGAAGGAGGATATCTGACTGTGGGCTACAC
	GTTCTACCGCACTCGATTCGAGATCCTGCCCAAGGGTCCAAACAGGGCGACTGTCCGAAAGAGCATTATC
	TACAATGTGAGAGAAGACTCTGCTGCTAATCTCTCTCTTGCCGCTGTCAATGAGAACCCAGATCAAGTTAA
	AGCCGTGGAGAACTTCCTCAGCGCAAACCCAAGCGCAAACCCGGGCTTCATCAACAATTGA
AcNCS14	ATGAAAGGGCAGCAGACCCACGAATACGTCGTGCCTTTCTCCGCGAGCGAGGTATGGGGAGTTTACAGC
	ACGCTCGAGCTCCCGCAGATCATCAAGAAAATGCCCAATTTCCTCGAGGACATTGAAGTCCACGGAGAC
	GGATCGGTGGGGACCCTCTTCACCCTCGTCGTTCCCCCAGGGGGGGCGTTCAGGATGTACAGAGAAAGGA
	TCGCGACACTGGACCACGCTAACCGGGTGAAGGAAGTCGACGTGGTCTCCGGCGGAGTGCTCGACATGG
	GCTACACCTTCTTCCGCACTCGATTCGAGATCGTTAACCTGGGTGCGACGGCCTCCATGATCAGATCCATC
	ATCATCTTCGAAATCACCGACGACAAGGCCTCCGACAACGTGAAGCTGGCGAACGTCACCGAAATGGAG
	CCCGTTGCCAAGGCCGTCGCGCAGTACCTGCTGGAGAAGAAAGCTTACCCGTCGAAGCTCCACGGGGAA
	CAGTCCTTCGAAATGGACGTCCCCCACTCCGCCGACGCCGTTTGGGACGTCTACAGCACGCTCAAGCTCC
	CCGAACTGTTCAAGGAACTCATTCCTGATATCAAATGGCAGGGAGATGGGTCTGTGGGAACCCTGTACTT
	CCTCTTCGCCGAACCATCCGACCCCATCGGGAACTACACAGAAAGGATCGCGACCCTGGACCACCAGAA
	GCGAATCAAGGAAGTCGACGTGATCGAAAACGGGGTACGGAACAAGGACTTCACCTTCTTCCGGACCCG
	GTTCGAGATCATCCCCAAGGGACCCAACGCGACCACCATCAGGTCCACGATCATATACGAGCTCAACCC
	GAAGTCCCCGACGGCCGCTACCAACATGCAGTTCGCGAATGTGATGGACATGAAGGGCGCCGGAGACGC
	CACTAGCAAGTACTTGAACAACCTGAACCAAGGTCGACCGGGGCCCGGCACCGGCTCTGGCGGGTCAGG
	TCCGCGGCCGTCCGGGGGTTGGGGCGAACCCGAGTTTCCCCCGCCGGCGGCGGGGGGTGGTGGAACCCA
	GCCACCTCCCACCGGCTCCTGGTGGTAG
AcNCS15	ATGAAAGGGCAGCAGACCCACGAATACGTCGTGCCTTTCTCCGCGAGCGAGGTATGGGGAGTTTACAGC
	ACGCTCGAGCTCCCGCAGATCATCAAGAAAATGCCCAATTTCCTCGAGGACATTGAAGTCCACGGAGAC
	GGATCGTTAGGGACCCTCTTCACCCTCGTCGTTCCCCCAGGGGGGGCGTTCAGGATGTACAGAGAAAGGA
	TCGCGACACTGGACCACGCTAACCGGGTGAAGGAAGTCGACGTGGTCTCCGGCGGAGTGCTCGACATGG
	GCTACACCTTCTTCCGTACTCGATTCGAGATCGTTAACCTGGGTGCGACGACCTCCATGATCAGATCCATC
	ATCATCTTCGAAATCACCGACGACAAGGCCTCCGACAACGTGAAGCTGGCGAACGTCACCGAAATGGAG
	CCCGTCGCCAAGGCCGTCGCGCAGTACCTGCTGGAGAAGAAAGCTTACCCGTCGAAGCTCCACGGGGAA
	CAGTCCTTCGAAATGGACGTTCCCCACTCCGCCGACGCCGTCTGGGACGTCTACAGCACGCTCAAGCTCC
	CCGAACTGTTCAAGAAACTCATTCCTGATATCAAATGGCAGGGAGATGGGTCTGTGGGAACCCTGCTCTT
	CCTCTTCGCCGAACCATCCGACCCGATCGGGAACTACACAGAAAGGATCGTGACCCTGGACCACCAGAA
	GCGAATCAAGGAAGTCGACGTGATCGAAAACGGGGTACGGAACAAGGACTTCACCTTCTTCCGGACCCG
	GTTCGAGATCATCCCCAAGGGACCCAACGCGACCACCATCAGGTCCACGATCATATACGAGCTCAACCC
	GAAGTCCCCGACGGCCGCTACCAACATGCAGTTCGCGAATGTGATGGACATGAAGGGCGCCGGAGACGC
	CACTAGCAACTACTTAAACGACCTGAACCAAGGTCGACCGGGGCCCGGCACCGGCTCTGGCGGGTCAGG
	TCCGCGGCCGTCCGGGGGTTGGGGCGAACCCGAGTTTCCCCCGCCAGCGACTGGGGGTGGGGGAGCCCA
	ACCACCTCCCACCGGCTCCTGGTGGTAG

Table S2. Accessions numbers for proteins used in phylogenetic tree construction.

NCS/PR10	**Speices**	**Protein name**	**Accession**
NCSs	*Nelumbo nucifera*	NnNCS1	ANI26411.1
		NnNCS3	ANI26412.1
		NnNCS4	ANI26413.1
		NnNCS5	AND61511.1
		NnNCS7	AND61512.1
	Papaver somniferum	PsNCS1	AAX56303.1
		PsNCS2	AAX56304.1
	Papaver bracteatum	PbNCS	ACO90258.1
	Argemone mexicana	AmNCS1	ACJ76785.1
		AmNCS2	ACJ76787.1
	Corydalis saxicola	CsNCS	AEB71889.1
	Coptis japonica	CjNCS2/CjPR10A	A2A1A1.2
	Thalictrum flavum	TfNCS	ACO90248.1
	Sinopodophyllum hexandrum	ShNCS	AIT42265.1
	Macleaya cordata	McNCS	OVA02905.1
	Prunus persica	PpNCS-like	XP_007221170.1
	Fragaria vesca	FvNCS-like1	XP_004307851.1
		FvNCS-like2	XP_004307852.1
	Cucumis sativus	CusNCS-like1	XP_004148088.1
		CusNCS-like2	XP_011650080.1
	Setaria italica	SiNCS-like1	XP_004956360.1
		SiNCS-like2	XP_004956358.1
		SiNCS-like3	XP_004979030.1
	Brachypodium distachyon	BdNCS-like	XP_003580488.1
	Zea mays	ZmNCS-like1	XP_008663543.1
		ZmNCS-like2	XP_008668968.1
	Cinnamomum micranthum	CmiCS-like	RWR89694.1
	Juglans regia	JrNCS-like	XP_018828970.1
	Aristolochia californica	AcaNCS-like1	XP_068634603.1
		AcaNCS-like2	XP_068669627.1
		AcaNCS-like3	XP_068634604.1
		AcaNCS-like4	XP_068634995.1
		AcaNCS-like5	XP_068635689.1
		AcaNCS-like6	XP_068635090.1
PR10 proteins	*Nelumbo nucifera*	NnPR10	XP_010278417.1
	Zea mays	ZmPR10	NP_001147373.1
	Glycine max	GmPR10	NP_001238280.2
	Vigna angularis	VaPR10	XP_017409824.1
	Picea glauca	PgPR10	AAF12810.1
	Pseudotsuga menziesii	PmPR10	AAF60972.2
	Sorghum bicolor	SbPR10	AAW83207.1
	Triticum aestivum	TaPR10	ACG68733.1
	Pinus pinaster	PpPR10	ADJ53040.1
	Picea glauca	PglPR10	ABA54791.1
	Pinus monticola	PmoPR10	AAL50001.1

Table S3. Amino acid sequence of NCSs used for phylogenetic tree construction without an accession number*.

Name	Amino acid sequence
CcNCS1	MYYFLEFFEKLDVIEGNGGVGTVLDIAFPPGAVPRSYKEKFVKVDHKNRLKEVVMIEGGYLDLGCTFYMDRIHVL<br>PKGANSCVIKSTLIYEIPDELVDSVGSLMSTEPLASMAKVISDYVLKQRKMTANKILRKELKTEMEVATSADSIWAV<br>YGSPDIPRLLRDVLLPGVFEKLDVIEGNGGVGTVLDIAFPPGAVPRTYKEKFVKVDHKNRLKEVVMIEGGYLDLGC<br>TFYMDRIHVLPKGPNTCVIKSTLIYEVPDEFADAVGSLISVEPLASMAEVISGYVLKQKKEAKKILRKELTHELEVPT<br>SADSIWAVYGSPDIPRLLRDVLLPGVFEKLDIVEGNGGVGTVLDIAFPPGAVPRSYKEKFVKVDHDKHLKEVVMIE<br>GGYLDLGCTFYMDRIHVLPKGPNSCVIESSLIYEVREELADVVGSLISIEPLASMAEVISSYVLKQQLRVFGVVVQPR<br>VGLSLLLCLILCLVILGGLLIGGVSI
PbNCS5	MMRKVIKYDMEVATSADSVWAVYSSPDIPRLLRDVLLPGVFEKLDVIEGNGGVGTVLDIAFPPGAVPRSYKEKFV<br>NIDRVKRLKEVIMIEGGYLDMGCTFYLDRIHVVEKTPSSCVIESSIVYEVEEEYADAMSKLITTEPLKSMAEVISNYVI<br>QKESVSARNIFNRQSVVKKEIRYDLEVPTSADSIWAVYSNPDIPRLLRDVLLPGVFEKLDVIEGNGGVGTVLDIVFPP<br>GAVPRRYKEKFVNINHEKRLKEVIMIEGGYLDMGCTFYLDRIHVVEKTSKSCIIKSSIVYEVKQECAEAISKLITTEPL<br>KSMAQVIANYVLKKQSVSDTNIPKKQSVLRKEITYETEVQTSVDSIWNVYSSPDIPRLLRDVLLPGVFEKLDVIAGN<br>GGVGTVLDIAFPLGAVPRRYKEKFVKINHEKRLKEVIMIEGGYLDMGCTFYMDRIHVLEKTPNSCVIESSIIYEVKEE<br>FADKMAKLITTEPLQSMAEVISAYVLRKRFEVFGLEIKQKLRYNLLLCLIICLVIAGGMLIGRVPL
CmaNCS1	MIEGGYLDMGCTFYMDRIHVVKKGPNSCVIASAIIYEVKEEFVDVVVPLITTEPLASMAEVISNYVLKKQRRVRKEL<br>TYEMEVPTSADSIWAVYSSHDIPRLLKEVLLPGVFEKLDVIEGDGGVGTVLDIAFPPGAVPRTYKEKFVKINHEKRL<br>KEVVMIEGGYLDMGCTFYMDRIHVLEKSPNSCVIESSIIYEVKEEFADVVGPLITTEPLASMSEVISNYVLKKQIRMF<br>GYVIKPKLGLSLLLCFILCLVLLGVLLIGGVPL
SdNCS1	MRKEVRYEMEVPTSADSIWAVYSSHDIPRLLKEVLLPGVFEKLDVIEGDGGVGTVLDIAFPPGAVPRTYKEKFVTIN<br>HEKRLKEVIMIEGGYLDMGCTFYMDRIHVLEKGPKSCIIASAIIYEVKEEFADVVVPLITTEPLASMAEVISNYVLKK<br>QRRVRKELTYEMEVPTSADSIWAVYSSHDIPRLLKEVLLPGVFEKLDVIEGDGGVGTVLDIAFPPGAVPRTYKEKFV<br>KINHEKRLKEVVMIEGGYLDMGCTFYMDRIHVLEKGPNSCVIESAIIYEVKEEFADVVVPLITTEPLASMAEVISNY<br>VLKKQIHVFGYVIKPKLGLSLLLCFILCLVLLGVLLIGGVPL
PsNCS3	MRKVIKYDMEVAVSADSVWAVYSSPDIPRLLRDVLLPGVFEKLDVIEGNGGVGTVLDIVFPPGAVPRSYKEKFVNI<br>DREKRLKEVIMIEGGYLDMGCTFYLDRIHVVEKTKSSCVIESSIVYDVKEECADAMSKLITTEPLKSMAEVISNYVIQ<br>KESFSARNILSKQSVVKKEIRYDLEVPISADSIWSVYSCPDIPRLLRDVLLPGVFEKLDVIEGDGGVGTVLDIVFPPGA<br>VPRSYKEKFVNIDREKRLKEVIMIEGGYLDMGCTFYLDRIHVVEKSLSSCVIESSIVYEVKEEYADAMSKLITTEPLKS<br>MAEVISNYVIQRESFSARNILNKNSLVKKEIRYDLEVPTSADSIWSVYSCPDIPRLLRDVLLPGVFQKLDVIEGNGGV<br>GTVLDIVFPPGAVPRSYKEKFVNINHEKRLKEVIMIEGGYLDMGCTSYLDRIHVVEKTSKSCIIKSSVVYEVKQECVE<br>AMSKLITTEPLKSMAEVISNYAMKQQSVSERNIPKKQSLLRKEITYETEVQTSADSIWNVYSSPDIPRLLRDVLLPGV<br>FEKLDVIAGNGGVGTVLDIAFPLGAVPRRYKEKFVKINHEKRLKEVVMIEGGYLDMGCTFYMDRIHVFEKTPNSC<br>VIESSIIYEVKEEYAGKMAKLITTEPLESMAEVISGYVLKKRLQVFGFEIKPKLRFNLLLCLIICLVIAGGMFVAGVPL

* The sequences were published in [47].

Table S4. Motif consensus of AcNCS proteins.

Name	*E*-value	Motif consensus
motif1	3.3E-338	NYKEKFVTLDHEKRVKEVDVIEGGVLDVGFTFYRTRFEIVEKGPNSTVIK
motif2	1.00E-196	MHGEQSFELEVPFPADDVWDVYSTLKLPELVK
motif3	1.60E-126	PPGWPFKMYRERIATLDHANRVKEVDVIEGGVLDYGYTFCRTRFEVVDQG
motif4	3.60E-94	QGDGGVGTILTVTLPPG
motif5	2.10E-87	MKGQRVHEYVVPFPAPDVWDVVGSLELPQJVKNMPGVLEDJTVHGDGSVG
motif6	2.80E-79	VSLVTLAQMEAVAKAVANYLAEKKSGPRK
motif7	6.30E-59	YELNPKSPTAAENMKLANVMIVKTAGDLTSDYLKNJNQ
motif8	3.70E-48	RSTILYEIDDEKASD
motif9	3.30E-11	GPGTGSGGSGPRPSGGWGEPEFPPPAAGGGGAQPPPTGSWW
motif10	3.10E-10	QKLPNIIEKID

Table S5. Primers used for qRT-PCR.

Gene name	Forward primer sequence (5'-->3')	Reverse primer sequence (5'-->3')
β-Actin	TTCAATGTCCCAGCCATGTACGTT	ACCGGAATCCAGTACAATACCAG
AcNCS1	GGGTTCGAATGCCTCGAGG	ACGGCGTTTATATTAGCCAG
AcNCS2	GGGTTCGAATGCCTCGAGG	TCAGCGAGATACTTGGCCAC
AcNCS3	GGCACCATTCTCGACATCTT	AGTCCGAAACCCAGTTCTTG
AcNCS4	GAAATTCGTGCATCATCAGG	TTCACCGAGATGCTGTGC
AcNCS5	GAGATTTGTGCATCATCAG	TTCACCGAGATGCTGTGC
AcNCS6/7	GAGTGAAGGTAGCGGAAGTG	ACGGGCGAACATTTGGAG
AcNCS8	GAGTGAAGGTAGCGGAAGTG	ACGGGCGAACATTTGGAG
AcNCS9	CGCAACTCGTCAAGAACATG	TCTCTGTACGTCTGAAATGGC
AcNCS10	ACGTTTGGGATGTCTACAGC	CCACCTTCTCTTTGTAGCTCG
AcNCS11	CAAACTCGACGGTGGTGAA	CTTCTCCTGGATAGGCGGA
AcNCS12	CAAACTCGACGGTGGTGAA	TATAGCCTCGGTACTCATAGC
AcNCS13	CAAATCTTCAAGACAGTGCCG	TCCTCGCTGTTCAATGTCAC
AcNCS14	GTTTGGGACGTCTACAGCAC	GCGAAGAGGAAGTACAGGGT
AcNCS15	GTCTGGGACGTCTACAGCAC	CGAAGAGGAAGAGCAGGGT

Table S6. Primers used for *AcNCS* cloning.

Primer name	Primer sequence (5'-->3')
AcNCS2-F	taagaaggagatatacatatgATGCGGGGGAAGAAGATCA
AcNCS2-R	tggtggtgctcgagtgcggccgcGCTTGGGGCGGCGG
AcNCS4/5-F*	taagaaggagatatacatatgATGCACGGTCAGGTCTCGC
AcNCS4/5-R*	tggtggtgctcgagtgcggccgcAGCGTCTGCATATCCGATCTCC
AcNCS14/15-F*	taagaaggagatatacatatgATGAAAGGGCAGCAGACCCA
AcNCS14/15-R*	tggtggtgctcgagtgcggccgcCCACCAGGAGCCGGTGGG

* The primers can be used to amplify two genes.
